# Prolonged ketamine infusion as a therapy for complex regional pain syndrome: synergism with antagonism?

**DOI:** 10.1111/bcp.12157

**Published:** 2014-01-22

**Authors:** Anthony E Pickering, Candida S McCabe

**Affiliations:** 1 School of Physiology & Pharmacology, University of BristolBristol, UK; 2 Faculty of Health and Life Sciences, University of the West of EnglandBristol, UK

Complex regional pain syndrome (CRPS) remains a troubling and often refractory pain condition that typically develops either following injury to a peripheral nerve (type II) or following trauma without obvious peripheral nerve damage (type I). It presents with a characteristic constellation of sensory, motor, autonomic and vascular signs, within which pain is the central feature. Its aetiology is uncertain [1], and there is an ongoing debate about whether it is primarily a neuropathic or an inflammatory/autoimmune condition [2].

The existing data from treatment trials of conventional analgesics, antineuropathic pain medications or nerve blocks have been largely disappointing [3,4], and much of the emphasis in treatment is on functional rehabilitation (see the recent Royal College of Physicians treatment guidelines [5]). The limitations of existing treatments means that a substantial cohort of CRPS patients (∼15 000 new cases per year in the UK based on the incidence figures of de Mos *et al*. [6]) is left with refractory, long-term severe pain causing significant disability. Consequently, there is a need to develop innovative treatment approaches, such as graded motor imagery, mirror visual feedback, spinal cord stimulation and transcranial magnetic stimulation, all of which have shown some utility in clinical trials. Set within this context, the review of Niesters *et al*. [7] in this issue of *BJCP* is timely, and articulates the arguments for a role of prolonged ketamine infusion in the management of neuropathic pain and specifically for CRPS.

## Ketamine

Ketamine is an anaesthetic drug that has caused polarizing tumult ever since its successful introduction to clinical practice in 1970 (reviewed by Domino [8]). Ketamine immediately found a role in battlefield anaesthesia during the Vietnam conflict, where its safety (because of relative lack of cardiorespiratory depressant effects) and its analgesic properties were advantageous. It was the first of the class of ‘dissociative’ anaesthetic agents and has remained an integral part of the clinical anaesthetic armamentarium for emergencies, for paediatrics and as a component of sedation for invasive medical procedures [9]. Since its earliest introduction it has also been a drug of abuse, taken for its hallucinogenic and euphoric actions (like phencyclidine). Within this setting, it causes both morbidity (e.g. bladder pathology) and mortality through overdose-related aspiration or psychotic delusions leading to self-harm [10].

The main molecular mechanism of action of ketamine was uncovered when it was found to be an antagonist at the NMDA class of glutamate receptor [11]. The NMDA receptor, then recently identified, was rapidly realized to be a molecular coincidence detector for neurones (fitting with the Hebbian postulate), forming a key player in pathways for synaptic plasticity, learning and memory [12]. Ketamine is a noncompetitive, use-dependent NMDA channel blocker that could prevent the induction of synaptic potentiation. It was also subsequently realized that NMDA receptors play a central role in the processes of induction and maintenance of pain sensitization, accounting for the analgesic efficacy of ketamine [13]. Although ketamine undoubtedly has actions at other relevant sites, including nicotinic and opioid receptors, as well as via monoamine reuptake transporters, it is likely that both the anaesthetic and the analgesic actions of ketamine are largely mediated by NMDA receptor antagonism [14]. Likewise, the psychototropic and sympatho-excitatory side-effects of ketamine are also predominantly mediated through NMDA receptor blockade.

From its earliest clinical use, there has been considerable interest in exploring the therapeutic potential of ketamine in new territories. It was used experimentally to model schizophrenia, where its propensity (like phencyclidine) to cause hallucinations and delirium was employed in human studies [15]. The recognition of its neuroprotective action led industry to use it as a template to develop other noncompetitive NMDA antagonists to treat the excitotoxicity of stroke, head injury and also chronic neurodegenerative conditions [16]. Although animal studies with pre-emptive administration of these NMDA antagonists supported a neuroprotective action, the subsequent clinical studies have failed to identify a significant benefit in most trials. Concerns have also arisen over potential neurotoxic actions of ketamine seen in some animal studies. Nonetheless, a similar rationale for the use of memantine (another noncompetitive NMDA antagonist) in Alzheimer's disease has seen the drug licenced for clinical use, because it can modify the progression of dementia [17].

Most recently, ketamine has attracted attention because it can produce an improvement in depressive symptoms in man after acute administration [18]. These antidepressant actions are causing a stir in the field because of the rapid onset of therapeutic effect (unique amongst the currently available medications), which may enable its use as an acute treatment of severe depression. Reverse translational studies in animals have suggested that this beneficial effect is the product of rapid synapse formation (within hours) within the cortex, which reverses the loss in synapse numbers seen in depression [19]. However, it is thought that ketamine is unlikely to be a suitable therapeutic agent in the longer-term treatment of depression because of its challenging pharmacokinetics and psychotomimetic side-effects. Hence, the search is currently underway for a better-tolerated NMDA antagonist that might be as efficacious for long-term administration in depression [20].

## The role of ketamine in pain management

Ketamine has an established place in the perioperative management of acute pain (at subanaesthetic doses <0.5 mg kg^−1^), where it has consistently been shown to be opioid sparing and to decrease nausea and vomiting while adding only a mild side-effect profile of its own [21]. The *S*(+) stereoisomer of ketamine has been shown to be twice as potent as an analgesic (mirroring its greater potency at the NMDA receptor). Ketamine acts by a distinct, synergistic route to opioids and has also been suggested to attenuate the development of opioid tolerance [22] and perhaps to prevent opioid-induced hyperalgesia. It therefore has an accepted role in balanced analgesic regimes and is deployed in cases where perioperative pain control is likely to be challenging.

A significant body of evidence has tested the attractive hypothesis that pre-emptive administration of ketamine might prevent the development of pain (both acute and chronic) by preventing the sensitization of neuronal circuits. Although positive findings were reported from animal studies, the data from human trials have not shown a clear pre-emptive benefit [23]. However, the acute analgesic effects of ketamine can outlast the expected half-life of the drug, suggesting that there may indeed be some suppression of neural sensitization in pain circuits [24].

Ketamine has also been employed in the treatment of chronic pain (particularly neuropathic pain), where it shows some short-term efficacy for refractory pain. However, its utility for chronic pain has been limited by its pharmacokinetics and side-effect profile. It has relatively poor bioavailability after oral dosing, and its short duration of action makes intermittent parenteral dosing impractical. Additionally, the psychotomimetic profile, nausea and vomiting, sympatho-activation, effects on cognitive function allied to risks of misuse and dependence give it a narrow therapeutic window. This has prompted efforts to explore the role of other NMDA antagonists with either better pharmacokinetics or NMDA receptor subtype selectivity, but these efforts have yet to yield a convincing alternative therapy. A recent meta-analysis of therapeutics for neuropathic pain did not find sufficient evidence to support a role for ketamine or the other NMDA antagonists [25].

## The role of ketamine in the treatment of complex regional pain syndrome

Despite these issues, several research groups have undertaken randomized, controlled trials and published thought-provoking papers on the use of prolonged ketamine infusions for treatment of refractory CRPS [26,27]. These researchers rationalize this approach on the basis that there may be a longer-lasting resetting of sensitized nociceptive circuits that can outlast the period of infusion (like that seen in acute pain [24]). The infusions were maintained at a subanaesthetic level and were either intermittently dosed over a 10 day period on an outpatient basis [26] or as a 5 day inpatient protocol [27]. These studies are credit-worthy because they have produced interesting affirmative findings and will have been challenging to undertake in a severe CRPS patient population. The analysis of these studies suggests that the prolonged infusions can produce reductions in pain that persist for weeks. Although issues with the quality of one of these trials [26] have been raised (see [28]), there would appear to be evidence of a substantial (>50%) analgesic effect that could be worthwhile in refractory cases of CRPS if its duration could be prolonged.

In a balanced consideration of these trials, it should be recognized that CRPS is a significant clinical problem with limited therapeutic options [5,29]. Any interventions capable of producing a lasting improvement in pain, motor function and disability merit serious consideration, and there are anecdotal reports suggesting that ketamine infusions are being deployed by pain clinicians globally for the treatment of a variety of pains, including CRPS. This raises the overarching question of whether this ketamine therapy can yet be advocated as an evidence-based, cost-effective, rational, safe and practicable therapy for CRPS.

Recent systematic reviews of treatments for neuropathic pain have not supported the use of any NMDA antagonist (e.g. [25]). However, CRPS type I is no longer considered to be a neuropathic pain syndrome according to the International Association for the Study of Pain definition [30]. Prolonged ketamine infusion has been included in the list of potential therapeutic options for the management of CRPS in the UK and Dutch guidelines, with both documents rating the evidence for this treatment as ‘moderate’ or ‘level 3’ [4,^5^,31] but the guidelines conclude that there is insufficient evidence to commend ketamine (amongst other drugs) as a part of routine pain clinical practice for the management of CRPS. This in part reflects the relatively short-term analgesic benefit and the requirement for repeated, prolonged hospital treatment along with the lack of improvement in affect or in functional capacity allied to enduring concerns about side-effects.

The beneficial effects of the ketamine infusion on pain scores are lost by 12 weeks, which must be set against the chronicity of the disease. Thus, repeated dosing would be needed for patients with CRPS. For these parenteral infusion protocols, this will require hospitalization and close observation and will have a cost implication; to date, no cost–benefit analysis has been published. An additional complication is that prolonged infusion of ketamine is known to cause hepatic dysfunction (see review [32]). This came to light when half of the subjects in a trial of repeated courses of ketamine infusions for CRPS developed elevated liver enzymes [33], resulting in the study being halted early. Although these elevations were slowly reversible, it clearly raises concerns about the safety of repeated dosing. It is also worth noting that liver toxicity has been reported in ketamine abusers, and this raises the spectre that the other side-effects, whether psychotomimetic, urinary or cognitive, seen in recreational users [10] would also become problematic in patients undergoing chronic ketamine treatment (see review [34]).

There is a suggestion from the authors (and see [35]) that prolonged dosing with ketamine may provide a greater likelihood of therapeutic benefit. This principle has been somewhat contentiously extended to the territory of prolonged ketamine coma (involving patients spending days anaesthetized and ventilated in an intensive care unit), which its advocates claim to be able to ‘cure’ chronic neuropathic pain; as yet, this is supported only by case reports and open-label, uncontrolled trials [36,37]. Ketamine coma, and indeed oral ketamine and ketamine combined with nerve block, are cited in the UK Guidance document under ‘Experimental research’ with insufficient evidence for their efficacy at present (Goebel *et al*. 2012) and are not included in the Dutch guidelines [31]. There are few centres or clinicians that would offer (on the available evidence) this as a reasonable therapeutic approach to the management of CRPS.

This need for chronic NMDA antagonist dosing also raises the issue of whether parenteral ketamine is the optimal drug for this purpose. An oral lozenge preparation of ketamine has been shown to have adequate pharmacokinetics [38] but is as yet unproven in any pain trial. Other studies of dosing with other NMDA antagonists, such as memantine and amantadine, have failed to show beneficial effects alone [3,4]. Case reports and a single study have reported some utility when memantine is used in combination with morphine and physical rehabilitation [39,40], although this may reflect an opiate-sparing effect or the benefit of physiotherapy techniques, rather than an analgesic action in its own right.

The apparent comparative lack of effectiveness of these other NMDA antagonists in CRPS may relate to their lower efficacy as antagonists or may also indicate that the ketamine is acting via another molecular mechanism. Niesters *et al*. [7] have started to investigate this possibility further, looking at influences on endogenous pain modulation and in imaging studies. Ketamine administration produces brain activity in areas that are part of pain modulation circuitry, such as the anterior cingulate, the insula and the brainstem, and in the same study they also report an increase in conditioned pain modulation in behaviour studies in volunteers (although they have also previously reported a suppression of another endogenous analgesic mechanism by ketamine, [41]). These effects could potentially also be mediated via the opioidergic or monoaminergic reuptake action of ketamine. Such actions to engage/modulate endogenous analgesia might also help to account for the long-duration ketamine effect, as this could potentially be self-reinforcing.

One of the most striking features of the ketamine infusion studies is the lack of improvement of mood or function despite the improvement in pain scores. This is surprising given the recent realization that ketamine exerts an antidepressant action [18]. It also runs counter to the belief that the functional deficits in CRPS are exclusively secondary to pain. This is more consistent with the idea that the motor disturbance is a separate pathological entity. It is perhaps also relevant that acute ketamine dosing is known to cause ataxia and motor impairment. Importantly, the current guidelines for the management of CRPS recommend an integrated interdisciplinary approach to the patient's care, which aims to reduce pain, restore function and improve quality of life [5,^29^,42]. Functional rehabilitation is at the centre of the care pathway and should be facilitated by education/self-management strategies, psychological and pharmacological support. Specifically, the risk–benefit profile of pharmacological interventions should be considered to ensure that they do not compromise engagement with functional rehabilitation, as for example, the excessive drowsiness that can be seen with opioids. Both of these ketamine infusion studies had cohorts with long disease durations, and therefore, secondary psychosocial consequences of pain are likely to have been prevalent. Future research is warranted to establish what additional benefit ketamine could provide within a multidisciplinary rehabilitation model with administration earlier in the disease progression.

Administering ketamine as an adjunct to physical therapies may also be advantageous (besides its analgesic action) because of the potential for increased cortical synaptic plasticity being ascribed to NMDA antagonists from the depression field. Notably, memantine was reported to facilitate reversal of cortical reorganization (in one subject) of the primary somatosensory map representing the painful CRPS limb [40]. Similar reports have been described for amputee phantom limb pains when memantine has been administered [43]. It is well established in phantom limb pains and CRPS that there is a correlation between levels of perceived pain intensity and the extent of cortical reorganization, with pain reducing as cortical maps normalize [43,44]. Changes in the primary sensory and motor maps in CRPS are thought to contribute to disrupted motor planning, altered sensory perceptions, referred sensations and body perception disturbances (see [45] for review). All of these clinically observed problems have shown some improvement with nonpharmacological strategies that have targeted correction, but perhaps these benefits could be further enhanced in the presence of NMDA antagonists.

The ketamine infusion studies have provided evidence of a clinically meaningful (albeit transitory) analgesic benefit; something that is all too rare in the pharmacological management of CRPS. However, the challenges posed by its pharmacokinetics and risk profile remain. The prospects for achieving similar outcomes using (hopefully) safer, more convenient, subtype-selective NMDA antagonists remains on the horizon [14,46], but for the present we have little evidence for effective alternatives. Perhaps we will need a change of mind set to consider the management of CRPS with ketamine as being akin to the management of cancer with chemotherapy, i.e. toxic, but if the outcome is sufficiently positive then it may justify the risks. To arrive at this point, we would need multicentre, randomized, controlled trials comparing best current management with/without ketamine infusion with long-term follow-up and outcomes (as per IMMPACT guideline [47]) judged on the basis of remission and survivor analysis balanced against the toxicity. The situation is therefore similar for other potential new therapies, such as spinal cord stimulation or intravenous immunoglobulins; a balance of risk *vs*. benefit. The evidence discussed above indicates that even after 40 years the ketamine tiger (to paraphrase Domino [8]) remains potent and still has teeth that merit a cautious approach to its use in the management of chronic pain.

## Competing Interests

All authors have completed the Unified Competing Interest form at http://www.icmje.org/coi_disclosure.pdf (available on request from the corresponding author) and declare: no support from any organization for the submitted work; no financial relationships with any organizations that might have an interest in the submitted work in the previous 3 years; no other relationships or activities that could appear to have influenced the submitted work.
